# Waking and Sleeping following Water Deprivation in the Rat

**DOI:** 10.1371/journal.pone.0046116

**Published:** 2012-09-24

**Authors:** Davide Martelli, Marco Luppi, Matteo Cerri, Domenico Tupone, Emanuele Perez, Giovanni Zamboni, Roberto Amici

**Affiliations:** 1 Department of Human and General Physiology, Alma Mater Studiorum-University of Bologna, Bologna, Italy; 2 Systems Neurophysiology Division, Florey Neuroscience Institutes, University of Melbourne, Melbourne, Australia; 3 Oregon National Primate Research Center, Oregon Health & Science University, Portland, Oregon, United States of America; Morehouse School of Medicine, United States of America

## Abstract

Wake-sleep (W-S) states are affected by thermoregulation. In particular, REM sleep (REMS) is reduced in homeotherms under a thermal load, due to an impairment of hypothalamic regulation of body temperature. The aim of this work was to assess whether osmoregulation, which is regulated at a hypothalamic level, but, unlike thermoregulation, is maintained across the different W-S states, could influence W-S occurrence. Sprague-Dawley rats, kept at an ambient temperature of 24°C and under a 12 h∶12 h light-dark cycle, were exposed to a prolonged osmotic challenge of three days of water deprivation (WD) and two days of recovery in which free access to water was restored. Two sets of parameters were determined in order to assess: i) the maintenance of osmotic homeostasis (water and food consumption; changes in body weight and fluid composition); ii) the effects of the osmotic challenge on behavioral states (hypothalamic temperature (Thy), motor activity, and W-S states). The first set of parameters changed in WD as expected and control levels were restored on the second day of recovery, with the exception of urinary Ca^++^ that almost disappeared in WD, and increased to a high level in recovery. As far as the second set is concerned, WD was characterized by the maintenance of the daily oscillation of Thy and by a decrease in activity during the dark periods. Changes in W-S states were small and mainly confined to the dark period: i) REMS slightly decreased at the end of WD and increased in recovery; ii) non-REM sleep (NREMS) increased in both WD and recovery, but EEG delta power, a sign of NREMS intensity, decreased in WD and increased in recovery. Our data suggest that osmoregulation interferes with the regulation of W-S states to a much lesser extent than thermoregulation.

## Introduction

Physiological regulation is known to be different during rapid eye movement sleep (REMS) when compared to non rapid eye movement sleep (NREMS) [1]. In particular, during NREMS a stable autonomic outflow is observed in the presence of a fully operant homeostatic control of physiological variables [1]–[4]. In contrast, during REMS a high variability of the autonomic outflow, which leads to large irregularities in arterial blood pressure, heart rate, and respiratory rhythm, is concomitant with an impairment of thermoregulation [1]–[4]. This impairment has been considered to be a visible consequence of a change in hypothalamic integrative activity, disabling, during REMS, the autonomic feedbacks sustaining body homeostasis [1]. Accordingly, Wake-Sleep (W-S) states change when a tonic maintenance of the hypothalamic regulation is needed, as it occurs during the exposure to a low ambient temperature (Ta). In these conditions, Wake increases, NREMS is variably affected and REMS is always reduced or even suppressed in proportion to the Ta levels [2], [4], [5], [6].

However, the recent finding from our laboratory that, following a central osmotic stimulation, the release of the antidiuretic hormone arginine vasopressin (AVP) was kept at the same levels in the different wake-sleep (W-S) states, indicates that hypothalamic osmoregulation is not impaired during REMS [7]. This suggests that the change in hypothalamic integrative activity in this sleep stage should concern structures related to thermoregulation, rather than the whole hypothalamus as previously hypothesized [1].

Since clarification regarding the issue of specificity of the impairment in physiological regulation during REMS may be relevant for the understanding of this sleep state, we sought to test the observed independence of osmoregulation from autonomic changes in sleep. To this end, REMS occurrence and overall W-S regulation were assessed in rats during exposure to a three-day water deprivation (WD) protocol. This condition is known to constitute an osmotic challenge leading to the progressive engagement of the whole set of mechanisms maintaining body fluid homeostasis [8], [9]. Moreover, since a REMS rebound was observed during the recovery (R) period following cold exposure [5], [6], [10], [11], [12], [13], W-S assessment was continued for two days after water was once again made freely available. Basically, two sets of parameters were assessed: i) those concerning the maintenance of osmotic homeostasis (water and food consumption; changes in body weight and fluid composition); ii) those concerning the effects of the osmotic challenge on behavioral states (hypothalamic temperature, motor activity, and W-S states).

To our knowledge no systematic W-S studies have been carried out so far during a prolonged osmotic challenge. However, some past observations showed that disturbances in body fluid metabolism such as WD [14] or the infusion of AVP in humans [15] decreased the time spent in REMS, while the central administration of AVP increased Wake in rats [16].

The results of the present study showed that in spite of a strong activation of the osmoregulatory mechanism, REMS occurrence was only mildly depressed during WD. In contrast, NREMS increased, but it was characterized by a reduction in the power density of the delta band (DPW) of the EEG (0.75-4.0 Hz), which is considered to be the major index of intensity of this sleep stage [17]. These changes were mirrored by a decrease in Wake. Wake-sleep modifications mainly concerned the dark period of the light-dark (LD) cycle.

## Materials and Methods

### Animals and experimental protocol

Seventy-one adult male Sprague-Dawley rats (Charles River, weight: 290–310 g) were used. Animals were housed in normal laboratory conditions: free access to food and water, Ta 24.0±0.5°C, 12-hour∶12-hour LD cycle (L: 09:00–21:00; 100 lux at cage level). The experiments were carried out according to the European Union Directive (86/609/EEC) and were under the supervision of the Central Veterinary Service of the University of Bologna and the National Health Authority.

The experimental protocol consisted in at least one day of baseline (BL), three days of WD and two days of R in which water was made available again. All other ambient conditions were kept constant. Changes of experimental phases were coincident with L onset.

### Determination of the parameters concerning the maintenance of osmotic homeostasis

#### i) Consumption of food and water, changes in body weight

Nine rats underwent the experimental protocol in a metabolic cage (Tecniplast) situated in a thermoregulated and sound-attenuated room. The water bottle and food box were placed on top of two plexiglas tubes (functioning as reservoirs for the collection of wasted food and water), which, in turn, were positioned on top of two precision balances (Sartorius LP820) in order to provide continuous weighing of food and water, thus accurately measuring consumption. Food was powdered to avoid hoarding. Data from both balances were sent every 20 s to a PC, where they were stored for off-line analysis. Animals were weighed at L onset.

#### ii) Collection of blood, cerebrospinal fluid and urine; determination of osmolality and electrolyte concentration in collected fluids

At the end of each of the different 24-h periods, fluids were collected, between 09.00–11.00, from 54 animals randomly selected from different groups which were kept in the described experimental conditions. After determining the volume, a sample of urine was stored at –80°C. Animals were anaesthetized (diazepam, Valium Roche, 5 mg/kg intramuscular; ketamine-HCl, Ketalar, Parke-Davis, 100 mg/kg intraperitoneal) to collect: i) blood from heart (4 ml in Li-heparine coated tubes, APTACA); ii) CSF from the cisterna magna (200 µl), according to Waynforth [18]. Hematocrit was determined by tube (Brand) centrifugation (3000×g for 15 min at 4°C). Plasma and CSF samples were stored at −80°C. Osmolality was measured, in undiluted samples, by means of a vapour-pressure osmometer (Delcon), while Na^+^, K^+^ and Ca^++^ concentrations were determined by means of an acetylene flame atomic-adsorption spectrophotometer (Solaar) in samples which were appropriately diluted with water acidified with HCl 1 M. For Ca^++^ determination samples were diluted with a 10 mM LaCl_3_-50 mM HCl solution [19].

### Determination of the parameters concerning the effects of the osmotic challenge on behavioral states

#### i) Recording of electroencephalogram, hypothalamic temperature and motor activity

While under deep general anaesthesia (diazepam, Valium Roche, 5 mg/kg intramuscular; ketamine-HCl, Ketalar, Parke-Davis, 100 mg/kg intraperitoneal), eight animals were implanted epidurally with two stainless-steel electrodes for frontal-parietal EEG recording. Furthermore, a thermistor mounted inside the tip of a stainless-steel needle (21G) was positioned above the left anterior hypothalamus to measure hypothalamic temperature (Thy). Since it has been shown that rectal and hypothalamic temperature follow the same time course in rats bearing cranial electrodes [20], we took changes in Thy as a good index of changes in core temperature. Plugs to connect EEG electrodes and the thermistor to the recording apparatus were embedded in acrylic dental resin (Res-Pal) anchored to the skull by small epidural stainless-steel screws implanted at the outer limit of the surgical field. Motor activity (MA) was monitored by means of a passive infrared detector (Siemens, PID11, Munich, FRG) placed on top of the recording cage.

Animals were allowed to recover from surgery for at least one week, while adapting to the recording apparatus in individual Plexiglas cages, kept in a thermoregulated and sound-attenuated box. At the end of R, they underwent the experimental conditions described above. Two days were used for the BL recording. Recordings were continuous throughout the experiment, with the exception of a 09:00 to 09:15 interval during which bedding, food and water were changed.

Data were handled by a user software (QuickBASIC, Microsoft, CA, USA) developed in our laboratory. The EEG signal was amplified (amplification factor: approximately 7000), filtered (high-pass filter: −40 dB at 0.35 Hz; low-pass filter: −6 dB at 60.0 Hz) and, after analog-digital conversion (sampling rate: 128 Hz), was stored on a personal computer (486/100 DX-4). The EEG signal was subjected to online fast Fourier transform, and EEG power values were obtained for 4-s epochs in the delta (DPW: 0.75–4.0 Hz), theta (TPW: 5.5–9.0 Hz) and sigma (SPW 11–16 Hz) bands. Thy signal was amplified (1°C/1V) before AD conversion (sampling rate: 8 Hz). MA signal was amplified and integrated before analog-digital conversion (sampling rate: 8 Hz) in order to make the output proportional to the amplitude and duration of movement (MA intensity). This system detected most of the movements related to the normal behavior of the rat, such as exploring, grooming, feeding and small movements during muscle twitching or brief awakenings in either NREMS or REMS.

The methods for the determination of the W-S stages and their parameters have been described previously [5]. Briefly, the analysis comprised two steps. The first consisted in visually scoring REMS episodes for the definition of the following parameters: total time amount, number and duration of episodes, duration of interval between episodes. The time length for the minimal duration of a REMS episode, as well as of a REMS interval between episodes, was fixed at 8 seconds [10], [11]. In the rat, REMS intervals have a bimodal distribution and the two interval populations are separated by a 3 min duration class [10], [11]. On this basis, two types of REMS were identified: single REMS, consisting of episodes preceded and followed by long REM sleep intervals (>3 min) and sequential REMS, consisting of episodes separated by short REM sleep intervals (≤3 min) and occurring in clusters. In the rat, the rate of occurrence of sequential REMS episodes constitutes the main factor of change for the amount of REMS [10], [11]. The second step consisted in the recognition of Wake and NREMS episodes by means of an automatic procedure developed and validated in our laboratory [5].

### Statistical analysis

A repeated-measures ANOVA was performed for: i) food and water consumption; ii) the time spent in the different W-S stages; iii) the number and duration of episodes; iv) Thy levels; v) power densities for EEG spectra. A one-way ANOVA was performed for: i) weight of animals; ii) hematocrit; iii) diuresis; iv) osmolality and electrolyte concentration values. A number of pre-planned non-orthogonal comparisons were made by means of the modified test, associated with the “sequential” Bonferroni [21] correction of the significance level [22]. These were performed for: i) differences between values of each treatment day (WD and R) and average BL values; ii) differences between L and D values.

## Results

### Maintenance of osmotic homeostasis parameters (consumption of food and water, body weight and body fluids composition)

**Figure 1:**
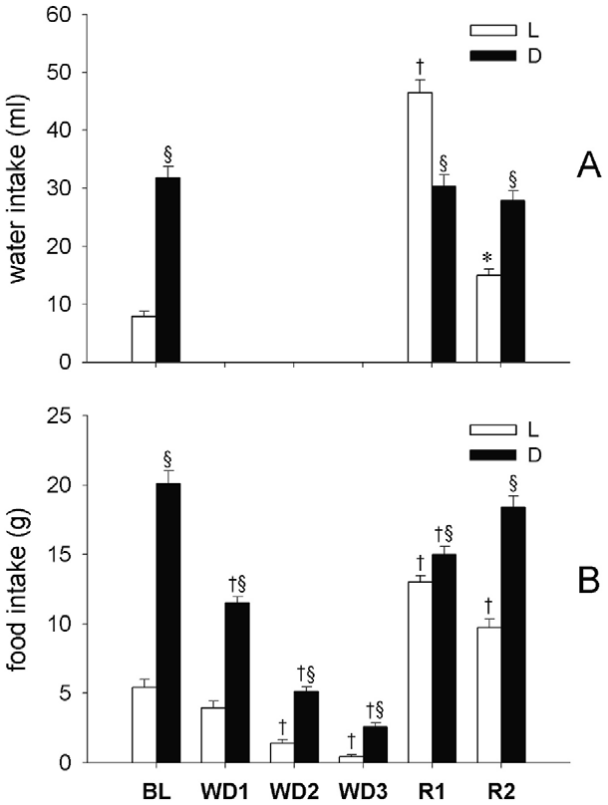
Water and food intake. Water (ml) and food (g) intake in rats (n = 9) under normal laboratory conditions (one day of baseline, BL), during a three-day water deprivation protocol (WD1, WD2, WD3), and in the two days of recovery (R1, R2) which followed the reestablishment of access to water. Values (means ± S.E.M.) for the 12-h Light (L, white bars) and the 12-h Dark (D, black bars) periods are shown. Statistically significant comparisons are shown: treatment *vs*. BL (*, P<0.05; †, P<0.01); D *vs*. L (§, P<0.05).

The levels of food and water intake during the experiment are shown in Figure 1. Both intakes were significantly larger during the D period than during the L period in BL (P<0.05). During WD, a progressive decrease in food intake was observed (L period: WD2 *vs*. BL, P<0.01; WD3 *vs*. BL, P<0.01; D period: WD1 *vs*. BL, P<0.01; WD2 *vs*. BL, P<0.01; WD3 *vs*. BL, P<0.01), although the usual L-D rhythm was maintained (P<0.05, for the three WD days). A rebound of both food and water intake was observed in R1 and R2 during the L period (R1 *vs*. BL, P<0.01; R2 *vs*. BL, P<0.01; for both), but not during the D period. In particular, during R1 a decrease in food intake was observed during the D period when compared to BL (P<0.01), leading to an attenuation of the normal L-D rhythm, while a significant reversal (P<0.01) of the normal L-D rhythm was observed for water intake.

A progressive decrease of body weight was observed during WD (indicated as means ± S.E.M; BL, 302±4 g; WD1, 274±4 g; WD2, 258±3 g; WD3, 244±3 g; P<0.01, for each WD day *vs*. BL), which was promptly recovered during R1 (R1, 292±4 g; R2, 299±4 g).

Changes in diuresis and urinary parameters across the experiment are shown in Table 1. During WD diuresis was progressively reduced to about 13% of normal BL levels (WD1 *vs*. BL, P<0.05; both WD2 and WD3 *vs*. BL, P<0.01). These changes were associated with an increase in urine osmolality, which reached statistical significance when compared to BL during both WD2 and WD3 (P<0.01). Also, large changes in urine electrolytes were observed during either WD or the R period when compared to BL: i) Na^+^ levels increased in WD1 (P<0.05) and WD2 (P<0.01) and decreased in R1 (P<0.01); ii) K^+^ levels increased in WD2 (P<0.01) and WD3 (P<0.01) and decreased in R1 (P<0.01); iii) Ca^++^ almost disappeared during WD2 and WD3, while its levels significantly increased during R2 (P<0.01).

**Table 1:** Diuresis and urinary parameters.

	BL	WD1	WD2	WD3	R1	R2
Diuresis (ml/24 h)	12.4±1.1 (9)	8.8±0.6 (9)*	2.6±0.4 (9)†	1.6±0.2 (9)†	11.5±1.7 (9)	13.6±1.1 (9)
Osmolality (mmol/Kg H_2_O)	1811±143 (9)	2269±149 (8)	3766±162 (9)†	4322±237 (9)†	1766±226 (9)	1864±153 (9)
Sodium (mmol/L)	219.5±15.5 (9)	296.2±16.4 (8)*	324.0±18.2 (9)†	265.4±23.0 (9)	56.2±10.5 (9)†	225.6±16.2 (9)
Potassium (mmol/L)	361.4±18.5 (9)	400.4±14.0 (8)	455.7±16.9 (9)†	425.1±12.2 (9)*	279.0±24.7 (9)†	335.0±16.2 (9)
Calcium (mmol/L)	0.201±0.065 (8)	0.086±0.038 (7)	0.018±0.003 (8)	0.026±0.005 (8)	0.407±0.090 (8)	0.841±0.172 (8)†

Daily diuresis, urine osmolality, and Na^+^, K^+^ and Ca^++^ urinary concentration in rats under normal laboratory conditions (one day of baseline, BL), during a three-day water deprivation protocol (WD1, WD2, WD3), and in the two days of recovery (R1, R2) which followed the reestablishment of the access to water. Values are expressed as means ± S.E.M. Number of cases are indicated between brackets. Statistically significant post-hoc comparisons *vs*. BL, are shown: *, P<0.05; †, P<0.01.

As shown in Table 2, hematocrit, plasma osmolality and CSF osmolality progressively increased during WD compared to BL levels. However, while hematocrit was significantly higher during the whole WD period (P<0.01, for each of the three WD days *vs*. BL), the increase in osmolality was statistically significant only in WD2 (P<0.05) and WD3 (P<0.01) for CSF and in WD3 (P<0.01) for plasma. All parameters quickly returned to BL levels during R1. No statistically significant variations in individual electrolytes were observed in either plasma or CSF.

**Table 2:** Blood, plasma and cerebrospinal fluid parameters.

	BL	WD1	WD2	WD3	R1	R2
***Blood***						
Hematocrit (%)	43.4±0.5 (10)	47.0±0.9 (10)†	52.8±0.8 (9)†	55.4±0.8 (10)†	44.6±0.7 (8)	44.8±0.6 (7)
***Plasma***						
Osmolality (mmol/Kg H_2_O)	293±5 (10)	301±2 (10)	303±5 (8)	310±3 (10)†	291±4 (7)	293±3 (8)
Sodium (mmol/L)	155.6±2.4 (10)	152.9±2.4 (10)	157.3±2.9 (8)	157.8±3.1 (10)	149.3±4.9 (7)	155.9±2.8 (8)
Potassium (mmol/L)	4.6±0.1 (10)	4.3±0.3 (10)	4.4±0.2 (8)	4.3±0.2 (11)	4.6±0.1 (7)	4.8±0.1 (8)
Calcium (mmol/L)	1.89±0.12 (10)	2.03±0.08 (10)	2.01±0.15 (8)	1.92±0.07 (10)	1.81±0.13 (7)	1.78±0.12 (8)
***Cerebrospinal fluid***						
Osmolality (mmol/Kg H_2_O)	297±6 (6)	305±2 (8)	311±3 (9)*	319±2 (7)†	300±3 (7)	301±1 (6)
Sodium (mmol/L)	161.3±10.8 (7)	174.7±4.3 (6)	173.0±3.1 (8)	177.0±4.2 (7)	165.0±3.1 (6)	169.2±2.0 (6)
Potassium (mmol/L)	4.9±0.7 (8)	4.0±0.3 (8)	4.0±0.4 (7)	4.7±0.9 (8)	5.2±0.9 (6)	5.3±0.7 (6)
Calcium (mmol/L)	0.98±0.07 (7)	1.07±0.05 (6)	1.05±0.08 (6)	1.03±0.07 (6)	0.97±0.04 (6)	1.03±0.04 (5)

Hematocrit, plasma omolality, and Na^+^, K^+^ and Ca^++^ concentrations in plasma and cerebrospinal fluid in rats under normal laboratory conditions (one day of baseline, BL), during a three-day water deprivation protocol (WD1, WD2, WD3), and in the two days of recovery (R1, R2) which followed the reestablishment of the access to water. Samples were collected at the end of each of the indicated 24-h periods, between 09.00–11.00. Values are expressed as means ± S.E.M. Number of cases are indicated between brackets. Statistically significant post-hoc comparisons *vs*. BL, are shown: *, P<0.05; †, P<0.01.

### Effects of the osmotic challenge on behavioral parameters (motor activity, hypothalamic temperature, wake-sleep stages, EEG power)

**Figure 2:**
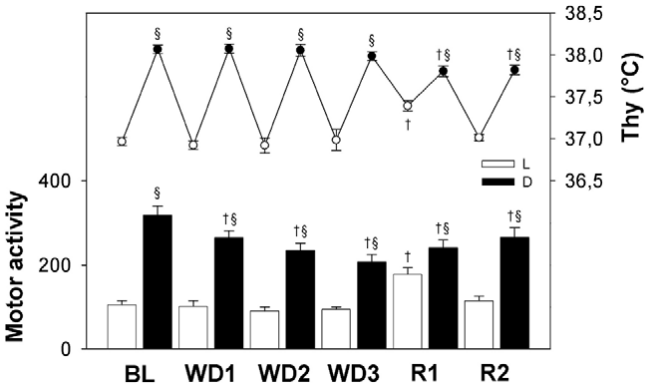
Motor activity and hypothalamic temperature. Motor activity (MA; bars referring to the left axis) and hypothalamic temperature (Thy, °C; dots referring to the right axis) in rats (n = 8) under normal laboratory conditions (average of two days of baseline, BL), during a three-day water deprivation protocol (WD1, WD2, WD3), and in the two days of recovery (R1, R2) which followed the reestablishment of access to water. Values (means ± S.E.M.) for the 12-h Light (L, white symbols) and the 12-h Dark (D, black symbols) periods are shown. Statistically significant comparisons are shown: treatment *vs*. BL (*, P<0.05; †, P<0.01); D *vs*. L (§, P<0.05).

The time course of MA and Thy during the experiment is shown in Figure 2 with a 12-h resolution. A clear daily oscillation was observed for both MA and Thy. During WD, MA but not Thy levels progressively decreased when compared to BL in the D period (P<0.01, for each of the three WD days *vs*. BL, for both variables). During the 1^st^ day of R, a large increase in both MA and Thy levels was observed during the L period (P<0.01, *vs*. BL, for both variables). This was mainly due to an enhancement in exploratory, drinking, and feeding behavior in the 4-h period which immediately followed the re-establishment of the access to water. Both MA and Thy decreased during the following D period when compared to BL levels (P<0.01, for both variables) leading to a dampening of the normal L-D rhythm. During R2, both MA and Thy levels returned to BL levels during the L period, but remained at a lower level when compared to BL (P<0.01, for both variables) during the following D period.

The time spent in different W-S states across the experiment is shown with either a 24-h or a 12-h resolution in Table 3. During the three days of WD a progressive decrease in the 24-h amount of Wake was observed (P<0.01 *vs*. BL, for each WD day) which was paralleled by an increase in the time spent in NREMS (P<0.01 *vs*. BL, for each WD day). Both effects were limited to the D period (P<0.01 *vs*. BL, for each WD day, for both), while no significant changes were observed during the L period. A slight progressive decrease in the time spent in REMS was also observed, which was significant only during WD3 when compared to BL (P<0.01). Sequential REMS, but not single REMS, was significantly affected during WD3 (P<0.01, *vs*. BL).

**Table 3:** Time spent in different Wake-Sleep states.

		BL	WD1	WD2	WD3	R1	R2
***24 h***							
**Wake**		51.6±0.7	47.2±1.0 †	46.6±1.1 †	45.8±0.9 †	54.3±1.6 *	47.8±1.3 †
**NREMS**		41.1±0.6	45.7±1.0 †	46.6±1.1 †	48.2±1.0 †	36.6±1.5 †	43.9±1.2 †
**REMS**		7.3±0.3	7.1±0.2	6.8±0.3	5.9±0.3 †	9.1±0.3 †	8.3±0.3 *
**Single REMS**		4.1±0.2	4.0±0.3	3.8±0.4	3.8±0.3	3.5±0.4	3.6±0.3
**Sequential REMS**		3.3±0.3	3.1±0.3	3.0±0.4	2.2±0.3 †	5.5±0.4 †	4.7±0.3 †
***12 h***							
**Wake**	**L**	27.1±0.6	25.8±1.5	23.9±1.1	26.8±1.5	46.9±2.4 †	30.2±1.7
	**D**	75.2±1.1 §	67.7±1.1 †§	68.4±1.1 †§	64.0±1.8 †§	61.5±2.5 †§	64.6±1.7 †§
**NREMS**	**L**	62.4±1.0	63.6±1.8	64.6±1.4	63.2±1.4	44.3±2.1 †	60.0±1.4
	**D**	20.6±0.9 §	28.7±0.8 †§	29.4±1.0 †§	33.9±1.6 †§	29.3±1.9 †§	28.5±1.7 †§
**REMS**	**L**	10.6±0.6	10.6±0.6	11.6±0.7	10.0±0.7	8.8±0.7	9.8±0.6
	**D**	4.2±0.5 §	3.7±0.6 §	2.2±0.2 §	2.0±0.4 §	9.2±0.9 †	6.9±0.6 *§
**Single REMS**	**L**	5.8±0.5	6.1±0.5	6.2±0.7	6.3±0.7	4.2±0.5	4.7±0.6
	**D**	2.4±0.3 §	2.0±0.4 §	1.4±0.3 §	1.3±0.3 §	2.9±0.4	2.6±0.3 §
**Sequential REMS**	**L**	4.7±0.4	4.5±0.6	5.3±0.8	3.7±0.5	4.7±0.6	5.1±0.6
	**D**	1.8±0.3 §	1.6±0.3 §	0.8±0.1 §	0.8±0.2 §	6.3±0.7 †	4.3±0.4 †

Time spent in the different Wake-Sleep states (Wake; NREMS, non-rapid eye movement sleep; REMS, rapid eye movement sleep) in rats (n = 8) under normal laboratory conditions (average of two days of baseline, BL), during a three-day water deprivation protocol (WD1, WD2, WD3), and in the two days of recovery (R1, R2) which followed the reestablishment of the access to water. Values (means ± S.E.M.) for the whole 24-h period or for the 12-h Light (L) and the 12-h Dark (D) periods are shown and expressed as the percent fraction of the 24-h or the 12-h period. Statistically significant comparisons are shown: treatment *vs*. BL (*, P<0.05; †, P<0.01); D *vs*. L (§, P<0.05).

**Figure 3:**
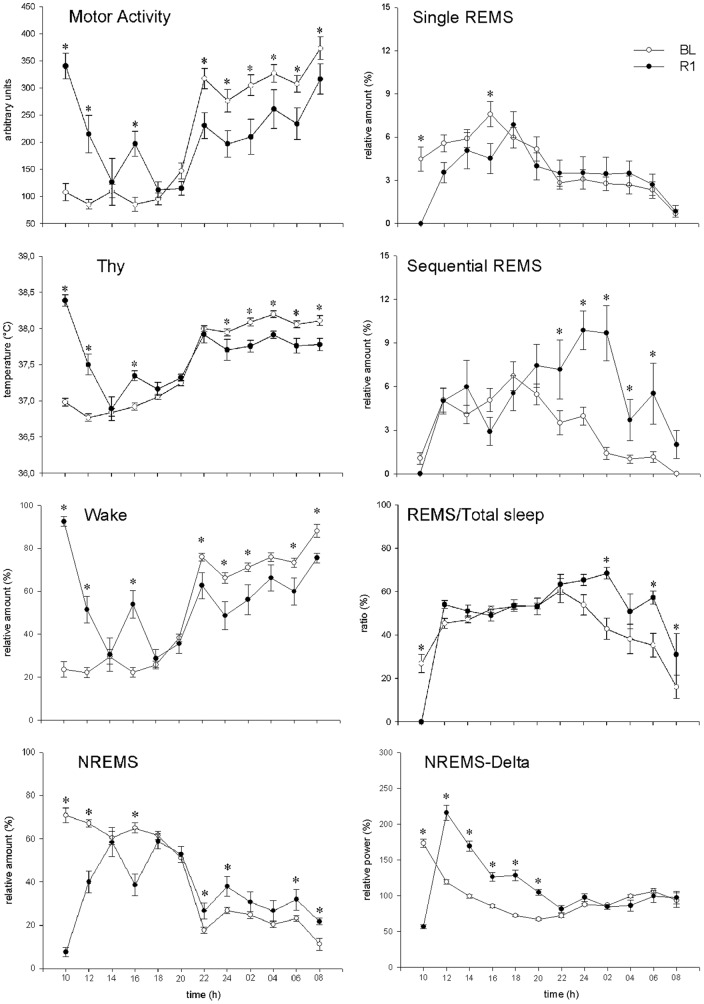
Comparison of the time course of changes in behavioral parameters on the first day of recovery with comparison to the baseline. Values (means ± S.E.M.) of different parameters are shown for each 2 h intervals of the L (09.00–21.00) and D (21.00–09.00) periods of the recovery (R1, filled circles) and base line (BL, empty circles): MA, motor activity; Thy, hypothalamic temperature. Values concerning sleep stages (Wake, NREM sleep (NREMS), REM sleep in the form of single (single REMS) and sequential (sequential REMS) episodes), the ratio of REMS to total sleep, levels of power density in the delta (0.75–4.0 Hz; band of the EEG during NREMS (NREMS-Delta)) are expressed as the percent fraction of each of the respective 2 h interval. Statistically significant comparisons are shown: treatment *vs*. BL (*, P<0.05).

As noted above, the 1st day of R was characterized by a rebound of ingestion behavior which affected wake-sleep occurrence. This is why behavioral parameters of R1 are analyzed in Figure 3 with a higher resolution (2-h) than in Table 3. The mean values of MA, Thy and Wake show a significant increase above control values that starts at the onset of the L period, the time of change in experimental protocol, and show a decline back to control values in the next 8 h. This pattern seems to affect these parameters during the following D period, since they follow the same time course as that observed in controls, but at significantly lower values. The overall NREM-REM sleep pattern is opposite to that of Wake, but it shows two significant peculiarities: i) a rebound of NREMS delta activity in the L period; ii) an increase of sequential REMS in the D period, which explains both the disappearance of REMS circadian rhythm in R1 and the shape of the time course of the REMS/Total Sleep Time (TST) ratio.

The 12 h resolution used in the analysis of W-S states reported in Table 3 for R2, shows that the overall pattern of change of W-S states observed in R1 in the D period is basically conserved in R2: a decrease in Wake, paralleled by an increase in NREMS (P<0.01 *vs*. BL, for both) and a large increase in the amount of sequential REMS (P<0.05 *vs*. BL).

The average number and duration of Wake and sleep episodes across the experiment are shown in Table 4. During WD, the number of both Wake and NREMS episodes was unaffected during the L period but progressively increased during the D period (P<0.01, *vs*. BL for each WD day, for both states), leading to the disappearance of the normal L-D rhythm. The concomitant decrease in the duration of the Wake episodes which was observed during the D period (P<0.01, *vs.* BL, for each WD day) was large enough to lead to the decrease in the 12-h amount of Wake previously described. On the contrary, the duration of NREMS episodes, which was only slightly reduced compared to BL, was enough to allow an increase in time spent in NREMS to occur. No substantial and consistent changes in the number and duration of either single or sequential REMS episodes were observed during WD.

**Table 4:** Number and duration of episodes of the different Wake-Sleep states.

		BL	WD1	WD2	WD3	R1	R2
**Wake**							
**Light**	No.	363.8±11.0	343.4±10.9	368.8±18.3	393.5±19.4	243.0±17.1 †	323.1±16.2
	Dur.	32±1	33±2	28±1	29±3	85±9 †	41±4
**Dark**	No.	220.8±10.9 §	322.3±12.8 †	380.4±22.5 †	444.0±31.6 †	231.3±16.9	231.4±15.9 §
	Dur.	153±11 §	90±5 †§	79±5 †§	65±6 †§	120±14 †§	122±10 †§
**NREMS**							
**Light**	No.	363.6±10.9	342.3±11.0	368.1±18.4	393.3±19.5	242.1±17.1 †	322.8±16.2
	Dur.	73±3	78±4	74±5	68±4	77±5	79±6
**Dark**	No.	220.0±10.8 §	321.8±12.9 †	380.4±22.3 †	443.5±31.6 †	231.1±16.8	231.1±15.8 §
	Dur.	41±2 §	39±1 §	34±2 §	34±3 §	55±2 *§	54±3 *§
**Single REMS**							
**Light**	No.	26.6±1.3	28.4±2.8	29.1±3.4	31.5±2.4	15.1±1.2 †	22.5±2.1
	Dur.	93±7	93±8	93±8	83±7	115±10 *	86±7
**Dark**	No.	12.0±1.6 §	10.4±2.1 §	6.3±1.1 §	6.9±1.6 §	13.4±1.2	12.3±1.6 §
	Dur.	88±3	86±4	94±6	92±12	93±8	95±7
**Sequential REMS**							
**Light**	No.	30.5±2.5	31.0±3.0	35.3±4.2	24.5±2.9	28.1±3.7	32.8±4.9
	Dur.	67±4	60±5	62±5	64±4	72±6	67±5
**Dark**	No.	10.7±1.6 §	9.4±1.7 §	3.9±1.0 §	4.1±1.1 §	37.1±4.5 †	24.6±2.1 †
	Dur.	76±2	76±4	100±12 †§	71±8	73±4	74±2

Number (No.) and duration (Dur., s) of episodes of the different Wake-Sleep states (Wake; NREMS, non-rapid eye movement sleep; REMS, rapid eye movement sleep) in rats (n = 8) under normal laboratory conditions (average of two days of baseline, BL), during a three-day water deprivation protocol (WD1, WD2, WD3), and in the two days of recovery (R1, R2) which followed the reestablishment of the access to water. Values (means ± S.E.M.) for the 12-h Light (L) and the 12-h Dark (D) periods are shown. Statistically significant comparisons are shown: treatment *vs*. BL (*, P<0.05; †, P<0.01); D *vs*. L (§, P<0.05).

During the first 12-h of R, a large increase in the duration (P<0.01, *vs*. BL) and a slight decrease in the number (P<0.01, *vs*. BL) of Wake episodes led to the increase in the 12-h amount of Wake previously described. This effect was paralleled by a decrease in the number of NREMS episodes (P<0.01, *vs*. BL). During the following D period, the duration of Wake episodes decreased (P<0.01, *vs.* BL) while that of NREMS episodes increased (P<0.05, *vs*. BL), leading to an increase in the 12-h amount of NREMS. This pattern was also observed during the D period of R2. For both Wake and NREMS, the normal L-D rhythm in the number of episodes disappeared during R1 but not during R2. The large increase in the number of sequential REMS episodes which was observed during the D period of both R1 and R2 (P<0.01, *vs*. BL) abolished the normal L-D rhythm of this parameter and led to the increase in the 12-h amount of REMS previously described. The number and duration of single REMS episodes were reciprocally affected during the L period of R1 (P<0.01, *vs*. BL, for both parameters), but this change did not affect the 12-h amount of REMS.

Average DPW during NREMS and TPW during either Wake or REMS are shown in Table 5 with a 12-h resolution and in Figure 3 with a 2-h resolution. Both NREMS-DPW and Wake-TPW were depressed by the WD procedure.

**Table 5:** Power density in the different band of the EEG during the different Wake-Sleep states.

	BL	WD1	WD2	WD3	R1	R2
**NREMS-DPW**						
**L**	102.4±0.5	101.2±1.4	84.5±2.5 †	78.1±2.2 †	145.3±5.4 †	91.4±4.6 *
**D**	93.1±1.4 §	76.1±3.1 †§	64.3±3.0 †§	58.9±3.2 †§	93.7±4.8 §	94.9±4.9
**Wake-TPW**						
**L**	102.1±1.1	106.9±3.4	99.3±3.4	94.8±3.8	111.9±3.8 *	99.8±5.3
**D**	99.2±0.5	95.2±2.6 §	88.4±3.1 †§	82.9±3.0 †§	99.7±4.5 §	96.3±4.2
**REMS-TPW**						
**L**	99.6±0.2	101.8±1.8	103.6±3.2	105.4±3.2	112.1±4.4 †	98.6±4.5
**D**	101.1±0.5	97.3±3.1	99.0±3.4	99.5±2.3	107.2±4.9	102.8±4.8

Relative levels of power density in the delta (0.75–4.0 Hz; DPW) band of the EEG during non-rapid eye movement sleep (NREMS) and in the theta (5.5–9.0 Hz, TPW) band of the EEG during either Wake or rapid eye movement sleep (REMS) in rats (n = 8) under normal laboratory conditions (average of two days of baseline, BL), during a three-day water deprivation protocol (WD1, WD2, WD3), and in the two days of recovery (R1, R2) which followed the reestablishment of the access to water. Values (means ± S.E.M.) for the 12-h Light (L) and the 12-h Dark (D) periods are shown and expressed as the percent of the mean 24-h level of the BL. Statistically significant comparisons are shown: treatment *vs*. BL (*, P<0.05; †, P<0.01); D *vs*. L (§, P<0.05).

As shown in Table 5, a statistically significant decrease compared to BL was observed for NREMS-DPW during both the L (P<0.01, for both WD2 and WD3) and the D (P<0.01, for each WD day) period and for Wake-TPW during the D period (P<0.01, for both WD2 and WD3), while a significant L and D rhythm of Wake-TPW appeared during the three days of WD (P<0.05, for each WD day). In contrast, no significant changes in REMS-TPW were observed during the WD protocol. During the R period, NREMS-DPW largely increased during the L period in R1 (P<0.01, *vs*. BL) and significantly decreased during the L period in R2 (P<0.01, *vs*. BL). This decrease led to the disappearance of the normal L-D rhythm. Both Wake-TPW and REMS-TPW significantly increased during the L period of R1 compared to BL (P<0.05, for Wake-TPW; P<0.01, for REMS-TPW). This increase led to the appearance of a significant L and D rhythm for Wake-TPW (P<0.05), but not for REMS-TPW, due to the maintenance of high levels of this parameter during the D period.

**Figure 4:**
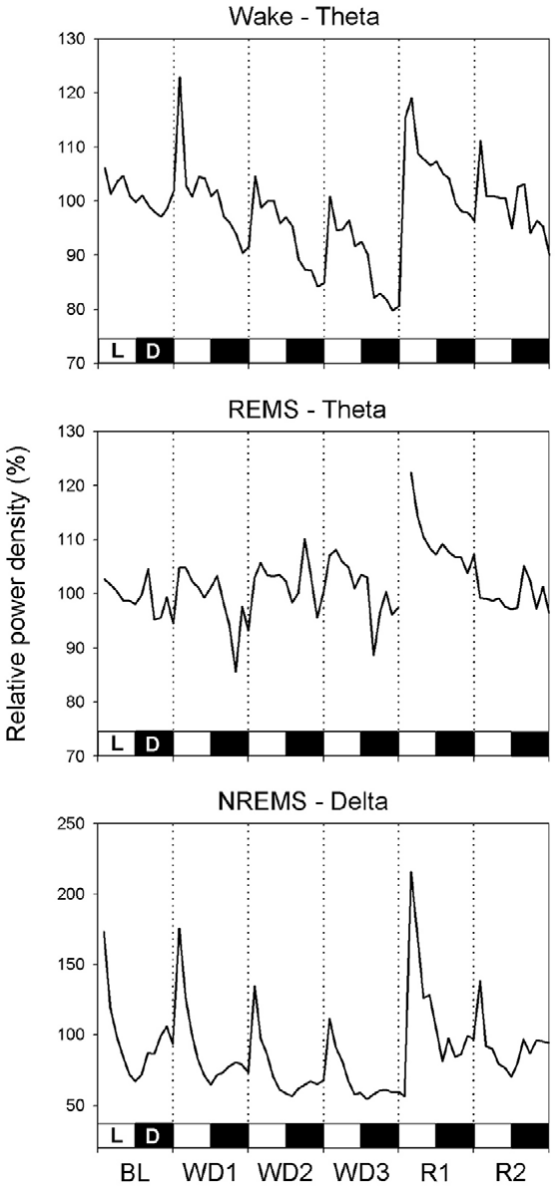
Power density in the different bands of the EEG during the different Wake–Sleep states. Relative power density in the delta (0.75–4.0 Hz; DPW) band of the EEG during non-rapid eye movement sleep (NREMS) and in the theta (5.5–9.0 Hz, TPW) band of the EEG during either Wake or rapid eye movement sleep (REMS) in rats (n = 8) under normal laboratory conditions (average of two days of baseline, BL), during a three-day water deprivation protocol (WD1, WD2, WD3), and in the two days of recovery (R1, R2) which followed the reestablishment of access to water. Values (means ± S.E.M.) are shown with a 2-h resolution and expressed as the percentage of the mean 24-h level of the BL. The 12 h∶12 h Light (L, white bars) – Dark (D, black bars) rhythmicity is shown.

**Figure 5:**
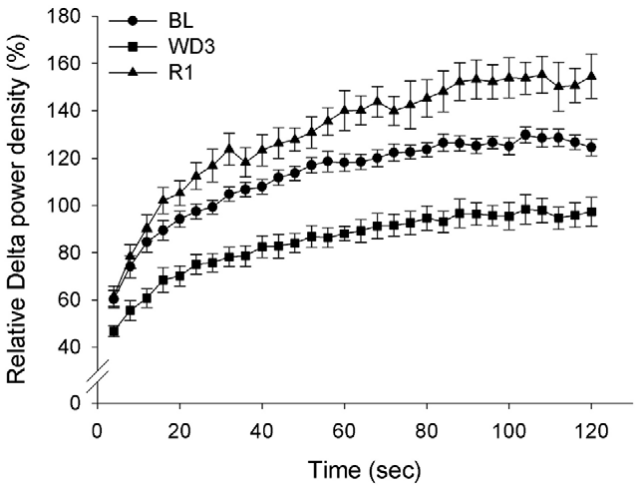
Build-up of delta power during consolidated non-rapid eye movement sleep episodes. Build-up of power density in the delta (0.75–4.0 Hz) band of the EEG during consolidated non-rapid eye movement sleep (NREMS) episodes (i.e. lasting more than 2 min) occurring in rats (n = 8) under normal laboratory conditions (average of two days of baseline, BL), during the third day of a water deprivation protocol (WD3) and the first day of recovery (R1) which followed the reestablishment of access to water. Values (means ± S.E.M.) are expressed as the percentage of the mean 24-h level of the BL.

The analysis of the power density time course in the three EEG bands with a 2-h resolution (Figure 4) showed that the peak of the NREMS-DPW, which occurs daily in the early L period, progressively decreased during the WD protocol. The peak was sharp during R1 but strongly depressed during R2. A sharp increase in the daily pattern for Wake-TPW appeared during the 2-h period immediately after the start of the WD procedure, followed by an apparently linear depression. This was not the case for REMS-TPW. Both Wake-TPW and REMS-TPW peaked within four hours after water was again made freely available, and progressively decreased during R1. The average build-up of NREMS-DPW during consolidated NREMS episodes (i.e. lasting more than 2 min) occurring during the L period is shown in Figure 5 for BL and for the two experimental days in which the average NREMS-DPW was maximally depressed (WD3) or enhanced (R1). The NREMS-DPW build-up appeared to be largely depressed during WD3 and enhanced during R1.

## Discussion

The results show that changes in the parameters of osmotic homeostasis and behavioral states follow different time and intensity pathways in WD and recovery. For this reason, the discussion will follow the experimental protocol and, in view of the main working hypothesis of an independence of sleep and osmolal regulations, changes concerning the cumulative duration and structure of W-S stages will be treated separately.

### Water deprivation

Changes in osmotic homeostasis were characterized by a large reduction in diuresis compared to BL values, by an overall stability of Thy and by changes in composition of the following body fluids: i) plasma: a significant increase in hematocrit (since the 1^st^ day), and osmolality (on the 3^rd^ day); ii) CSF: a significant increase in osmolality (on the 2^nd^ and 3^rd^ day); iii) urine: a relevant increase in osmolality, Na^+^/K^+^ concentration and an unexpected decrease in Ca^++^ concentration. Regarding the absolute excretion values (mmol/24 h), K^+^ and Na^+^ excretion decreased broadly in parallel, while Ca^++^ followed a much steeper downward slope. Taken together, these results show that in WD cation homeostasis in extracellular fluid is well preserved. Since we did not observe the sodium diuresis reported by others [23], it is likely that a stable cation concentration is achieved by a shift of intracellular water to the extracellular compartment. As far as Ca^++^ is concerned, it would appear that this mechanism is integrated by changes in either solubility, mediated by bone surface proteins [24], [25], or excretion threshold at kidney level [26].

Beside the wake-sleep cycle, behavioral parameters encompass ingestion and MA. Water deprivation is characterized by: i) a progressively large reduction in food intake (dehydration-induced anorexia) [27] which starts in the D period of the LD cycle on the 1^st^ day and then proportionally extends to both L and D periods on the 2^nd^ and 3^rd^ day; ii) a consistent reduction in MA in the D period of the LD cycle.; iii) a reorganization of the amount of W-S stages in the D period of the LD cycle, characterized since the 1^st^ day by an increase in NREMS and a parallel decrease in Wake and a non-significant decrease in REMS.

### Recovery from water deprivation

Diuresis was restored to the BL values while hypothalamic temperature significantly decreased below the BL in the D period of the LD cycle. The osmolality and cation content of plasma and CSF were restored to BL values. However, this stability corresponded to a significantly lower absolute urinary excretion of K^+^ and Na^+^ on the 1^st^ day, while BL values were re-attained only on the 2^nd^ recovery day. In the same time period, Ca^++^ excretion went from a two-fold to an almost five-fold increase.

Although a restoration of BL values was shown to be in progress, most behavioral parameters remained significantly different from their corresponding BL levels in the D period of both R days. In particular: i) MA remained lower than the BL; ii) the amount of Wake and NREMS remained respectively lower and higher than the BL. Two exceptions to this pattern are worth noting: i) in the L hours of the 1^st^ day of recovery, the time spent drinking significantly increased the amount of Wake and reduced the amount of NREMS; ii) the amount of REMS increased in the D period of both R days. Thus, the common pattern of behavioral results consisted of changes in both WD and R mainly in the D period of the LD cycle.

### Changes in the cumulative duration and structure of sleep stages

The results show an opposite pattern of change concerning the amount of sleep: the amount of NREMS increased during both WD and R, while REMS showed only a slight decrease in WD, and increased during R. The changes in the structure of sleep stages appear to be more subtle than those seen in the cumulative duration.

As far as NREMS is concerned, we observed a sharp contrast between its time course and the findings from EEG analysis. In fact, DPW, the hallmark of NREMS “intensity” in mammals [17], decreased during the WD period in both L and D hours, although it maintained its BL diurnal pattern. In the L hours of the 1^st^ day of recovery, the amount of NREMS was reduced by the time spent drinking, but DPW strongly increased in accordance with a “classical” recovery pattern following deprivation. This suggests that an accumulation of the need for slow-wave activity may follow not only the absence of NREMS, but also a selective absence of slow waves during NREMS [17]. Two lines of observations suggest that the dissociation between temporal and low EEG-frequency features of NREMS are not related to W-S processes. Firstly, the analysis of DPW accumulation in NREMS episodes lasting more than 2 min (consolidated episodes) suggests that the observed decrease in WD is not due to sleep fragmentation. In contrast to this, a significant decrease in DPW during NREMS caused by sleep fragmentation has been detected in rats exposed to a very low Ta [5]. Secondly, in WD the pattern of change in DPW in NREMS is concomitant with a constancy of TPW in Wake in the L period, or with its decrease during the D period. This is in contrast with the observation that, in the rat, sleep deprivation increases TPW in waking [12], [28]. Thus, it may be hypothesized that these changes may rather depend on dehydration-rehydration processes. Bearing this in mind, the fact that CSF concentration of principal cations did not significantly change in either WD or R rules out the possibility that these processes may induce a diffused alteration of cell excitability. This interpretation is further supported by the finding that theta power during REMS remains stable. The fact that the increase in the cumulative duration of NREMS is almost exactly paralleled by a reduction in Wake suggests that EEG changes in NREMS may be due to a differential influence of dehydration-rehydration processes on brain arousal systems [29].

The mild depression of REMS during WD reached statistical significance on the 3^rd^ day of this condition, because of the summing up of a reduction affecting both the L and D periods of the LD cycle. This result is consistent with recent observations from our lab showing that the responsiveness of hypothalamic structures to an acute osmotic challenge is not impaired during REMS [7]. Accordingly, it confirms that the impairment of thermoregulation observed during REMS [1] is not explicable on the basis of a broad impairment of hypothalamic integrative activity.

The reduction in the amount of REMS during WD concerned sequential episodes, that is, those mainly responsible for REMS modulation in the rat [5], [10], [11], [30], [31], [32], [33]. Accordingly, the small REMS rebound observed in R was due to an increase in the same kind of REMS episodes.

Solid experimental evidence shows that the homeostatic regulation of body fluids and temperature share regulatory networks at the hypothalamic and brainstem level [34], [35], [36], [37], [38]. The fact that during REMS osmoregulation is not impaired [7] while thermoregulation is [1], implies that the occurrence of a REMS episode is related to a functional dissociation between the hypothalamic control of thermoregulation and that of osmoregulation. Thus, it may be hypothesized that the sustained activity of the osmoregulatory network during WD may reduce the rate of occurrence of such a dissociation. This would more likely depress the occurrence of sequential REMS episodes since they are, by definition, separated by short intervals. In the same line of reasoning, the increase in sequential episodes during R suggests that this functional impairment has been rapidly reverted.

As shown by the higher resolution analysis employed for the 1^st^ day of recovery, the initial part of R was affected by the intervention of behavioral regulation of osmotic homeostasis, prompted by the reintroduction of free access to water. The efficacy of this regulatory behavior is shown by the restoration of normal diuresis, which was concomitant with an exceedingly high consumption of food and water in the L hours. Coherently with this regulatory behavior, values of MA, Thy and wake increased immediately to a maximum, decreased to control values in the next few hours, and further decreased below these levels in the D hours. This pattern of activity appeared to influence parameters concerning the amount of sleep in the L hours, which were characterized by values below baseline. In the D hours, the amount of NREMS and REMS followed a separate time course: i) NREMS returned to the same levels as those observed during the D hours of the previous WD; ii) REMS increased above these levels, as clearly underlined by the time course of the ratio of REMS to total sleep. The total rebound of REMS during the D hours of R was slightly larger than that expected from the previous loss [6]. A possible explanation of this result may be conveyed by the observation that, in the rat, an administration of food limited only to the L hours of the LD cycle caused an increase in REMS in the D hours [39], [40]. It may be noted that, in our experimental set, the passage from WD to water availability coincided with the onset of the L period of the LD cycle and that the dehydration anorexia was ended by the concomitant resumption of water and food.

On the 2^nd^ day of R, the overall course of these sleep changes was repeated, while that concerning the ingestion parameters moved toward baseline levels, almost restoring the usual L-D pattern. Although water deprivation may be considered a stressful condition, the findings concerning wake-sleep show that it neither increases the amount of arousal nor decreases the amount of sleep. Regarding this, it has been observed that the increase in plasmatic corticosterone concentration in WD is not driven by ACTH, but by circulating AVP [41], [42]. In line with these findings, in the compensatory drinking that follows water deprivation, it is the drop of AVP that quickly reduces corticosterone levels. Thus, it would appear that the principal peripheral effector of stress is regulated according to the specific homeostatic challenge represented by the maintenance of osmolality, rather than by the general intervention of the hypothalamic pituitary adrenal axis through CRH-ACTH.

### Conclusions

In normal laboratory conditions, the regulatory intervention of behavior is usually restricted, but waking and sleeping, which represent a basic set of behavioral states, are easily displayed. An exposure to low Ta, comparable for duration to the WD period used in this experiment, would have induced a relevant reduction in the occurrence of REMS, as a behavioral thermoregulatory response. Our data suggest that a tight osmolal homeostasis allows for a normal occurrence of the thermal unresponsiveness of preoptic hypothalamic structures which constitutes the pre-requisite to enter REMS. This may be particularly relevant for animals of small size which are exposed to quick changes in body temperature and have a limited amount of body water. Finally, since it is well known that the physiological control of body fluids evolved before that of body temperature, it may be hypothesized that REMS is closely related to homeothermia and metabolism.
